# An Improved Belief Entropy to Measure Uncertainty of Basic Probability Assignments Based on Deng Entropy and Belief Interval

**DOI:** 10.3390/e21111122

**Published:** 2019-11-15

**Authors:** Yonggang Zhao, Duofa Ji, Xiaodong Yang, Liguo Fei, Changhai Zhai

**Affiliations:** 1 Key lab of Structures Dynamic Behaviour and Control of the Ministry of Education, Harbin Institute of Technology, Harbin 150090, China; 18b933053@stu.hit.edu.cn (Y.Z.); yangxd@hit.edu.cn (X.Y.); zch-hit@hit.edu.cn (C.Z.); 2 Key lab of Smart Prevention and Mitigation of Civil Engineering Disasters of the Ministry of Industry and Information Technology, Harbin Institute of Technology, Harbin 150090, China; 3 School of Management, Harbin Institute of Technology, Harbin 150001, China; feiliguo@163.com

**Keywords:** Dempster-Shafer theory, uncertainty measure, Deng entropy, belief interval

## Abstract

It is still an open issue to measure uncertainty of the basic probability assignment function under Dempster-Shafer theory framework, which is the foundation and preliminary work for conflict degree measurement and combination of evidences. This paper proposes an improved belief entropy to measure uncertainty of the basic probability assignment based on Deng entropy and the belief interval, which takes the belief function and the plausibility function as the lower bound and the upper bound, respectively. Specifically, the center and the span of the belief interval are employed to define the total uncertainty degree. It can be proved that the improved belief entropy will be degenerated to Shannon entropy when the the basic probability assignment is Bayesian. The results of numerical examples and a case study show that its efficiency and flexibility are better compared with previous uncertainty measures.

## 1. Introduction

Uncertainty results from both the objective world and human’s subjective cognition, that is aleatory uncertainty and epistemic uncertainty [1]. The aleatory uncertainty is mainly caused by variance and randomness, which is closely associated with probability. Hence, the aleatory is ineluctable. The epistemic stems from the lack of knowledge. With the improvement of people’s knowledge level, the epistemic uncertainty can be reduced to some extent. Usually, uncertainty will cause negative effects and consequences for decision makers who attempt to take some principles to avoid risk [2,3]. Handling and reducing uncertainty hidden in information have always been a difficult problem to be resolved in various fields. Nevertheless, the measurement of uncertainty is of vital importance because quantifying the uncertain degree of certain information is the foundation and prerequisite before further information processing and fusing [4,5].

A physical quantity, called entropy, was initially proposed by Clausius to measure uncertainty in statistical thermodynamics [6]. Then, Shannon entropy [7] developed by Shannon was extended to solve the problem of measuring uncertainty under the probability theory and was proved effective for handling the uncertainty in some application systems [8,9,10]. Nevertheless, it does not produce desired results when measuring uncertainty of basic probability assignment (BPA) in Dempster-Shafer (D-S) theory that was put forward by Dempster [11] and then developed by Shafer [12]. The theory has proved to have significant advantages in representing, processing and fusing uncertain information or data by assigning a probability to the subsets of a set comprising multiple solutions rather than to each of the individual solution [13,14] and has been accepted as de facto standard in many fields [15,16,17,18,19,20,21,22,23,24,25,26,27,28,29,30,31,32,33,34], such as risk assessment [16,17,18,19], fault diagnosis [20,21,22,23], pattern classification [24,25,26,27], knowledge reasoning [28,29], and sensors’ network analysis [30].

Measuring uncertainty of BPA in the framework of D-S theory is always an open issue. Many efforts have been made to extend Shannon Entropy to measure uncertainty of BPA. There are two main perspectives for measuring uncertainty, namely discord [35,36] and non-specificity [37]. For the former, “Confusion” [38], “Dissonance” [39] and “Strife” [40] were introduced to measure or quantify uncertainty. In terms of the latter, a generalized Hartley entropy originally proposed by Dubois and Prade was employed to represent it [37]. Yager [39] and Korner [41] gave their definitions and methods to measure non-specificity. These methods only consider either discord or non-specificity when measuring uncertainty. However, a mass function is a generalized probability assigned on the power set of the frame of discernment (FOD) and a focal element of the FOD contains one or more events [42]. Hence, discord and non-specificity should be incorporated together to measure the uncertainty of BPA. In this way, Deng entropy [43] is put forward by taking total non-specificity and discord into consideration simultaneously based on Shannon entropy and attracts plenty of attentions [21,44,45].

The belief interval also provides a new insight to measure the uncertainty of BPA [46], which takes the belief function and the plausibility function as the lower bound and the upper bound, respectively. Based on the belief interval, a distance-based total uncertainty measurement was proposed [47] by Yang and Han under D-S theory framework. The average distance between the belief interval of each singleton and the most uncertain case is used to represent the total uncertainty degree. Then, Deng et al. [48] gave an improved method by calculating the distance between the belief interval and the so-called most uncertain interval to define the uncertainty measurement. Although this method makes up some deficiencies of conventional methods, it does not degenerate into Shannon entropy when the BPA is Bayesian. This is counterintuitive because D-S theory is considered as a generation of probability theory. Pan and Deng [46] proposed a new belief entropy to measure uncertainty of BPA considering belief function and plausibility function while ignoring the span of belief interval, which contains more information and variance. Wang and Song [49] provided an uncertainty measure AU which considers the imprecision of the belief interval. AU is more sensitive to the change of belief structures and has no computational burden. However, the monotonicity which is a crucial property of an uncertainty measure of AU is violated.

In this paper, an improved belief entropy is proposed based on Deng entropy and the belief interval. The improved belief entropy takes advantage of the central value and the span of the belief interval. It replaces the BPA in Deng entropy with the central value of the belief interval and adds a correction factor associated with the span of the belief interval which represents the imprecision of the belief interval. Several numerical examples and a case study about fault diagnose are employed to verify the effectiveness and applicability of the improved belief entropy.

The rest of this paper is organized as follows. Preliminaries of D-S theory and uncertainty measure are briefly introduced in Section 2. In Section 3, an improved belief entropy is proposed based on the belief interval and Deng entropy. Section 4 gives some numerical examples to verify the efficiency and flexibility of the improved belief entropy. In Section 5, a case study of fault diagnose is presented to show the applicability. Finally, conclusions are summarized in Section 6.

## 2. Preliminaries

### 2.1. D-S Theory

Several preliminaries are briefly illustrated [4,50].

Let Θ be a nonempty finite set of events or propositions that are mutually exclusive and exhaustive. Θ called frame of discernment (FOD) is defined as follows:

(1) $$\Theta =\{\theta_{1},\theta_{2},\ldots \theta_{i},\ldots ,\theta_{N}\}.$$

$2^{\Theta }$ called the power set of Θ is represented as:

(2) $$2^{\Theta }=\left\{⌀,\left\{\theta_{1}\right\},\left\{\theta_{2}\right\},\ldots ,\left\{\theta_{i}\right\},\ldots ,\left\{\theta_{N}\right\},\ldots \left\{\theta_{1},\theta_{2},\ldots ,\theta_{i}\right\},\ldots ,\left\{\Theta \right\}\right\}.$$

where *⌀* denotes the empty set. Each element in the power set Θ is called a hypothesis or proposition.

In the FOD Θ, a mass function also called BPA or the belief structure is defined as follows:

(3) $$m:2^{\Theta }\to \left[0,1\right].$$

BPA should meet the following conditions:

(4) $$m\left(⌀\right)=0,\sum_{A\in 2^{\Theta }}m\left(A\right)=1.$$

*A* is called a focal element when m(A)>0 and the set of all focal elements and their corresponding BPAs compose a body of evidences (BOEs). m(A) represents how strongly the evidence supports the proposition *A*.

The belief function and plausibility function are defined as follows, respectively:

(5) $$Bel\left(A\right)=\sum_{⌀\neq B\subseteq A}m\left(B\right),Pl\left(A\right)=\sum_{B\cap A\neq ⌀}m\left(B\right).$$

It is obvious that ∀A⊆Θ, Bel(A) <Pl(A). Bel(A) and Pl(A) represent the lower and the upper boundary of the degree that the evidence supports *A*. [Bel(A),Pl(A)] is considered as the belief interval for *A*.

In D-S theory, two evidences, denoted as $m_{1}$ and $m_{2}$, can be combined according to Dempster’s rule of combination [11,12] as follows:

(6) $$m\left(A\right)=\left(m_{1}⊕m_{2}\right)\left(A\right)=\frac{1}{1-k}\sum_{B\cap C=A}m_{1}\left(B\right)m_{2}\left(C\right).$$

where *k* is called the conflict coefficient which measures the degree of conflict of $m_{1}$ and $m_{2}$. If *k* = 0, it means that there is no conflict between $m_{1}$ and $m_{2}$. If *k* = 1, there is an absolute conflict. In other words, the greater *k* is, the higher the degree of conflict is. *k* is defined as follows:

(7) $$k=\sum_{B\cap C=⌀}m_{1}\left(B\right)m_{2}\left(C\right).$$

### 2.2. Shannon Entropy and Derivatives for D-S Framework

Shannon entropy, also known as information entropy and proposed by Shannon in 1948, is closely associated with uncertainty. Shannon referred to the concept of thermal entropy, which is a physical quantity indicating the degree of chaos of molecular states in thermodynamics. He thought that there was a close link between information volume and uncertainty, and then defined information entropy as follows:

(8) $$H_{s}=-\sum_{i=1}^{N}p_{i}\log_{b}p_{i}.$$

where $H_{s}$ denotes Shannon entropy (information entropy), *N* is the number of basic states, and $p_{i}$ meets $\sum_{i=1}^{N}p_{i}=1$. *b* is assigned a value of 2 when the unit of information is bit. Then, Shannon entropy can be expressed as follows:

(9) $$H_{s}=-\sum_{i=1}^{N}p_{i}\log_{2}p_{i}.$$

Great information entropy will contain more complexity and uncertainty in information so Shannon entropy succeeds in handling the problem of measuring uncertainty of information under the framework of probability theory to a large extent. Nevertheless, there are still some limitations [48]. The concept of entropy is also an open issue under the framework of D-S theory. Definitions of some typical uncertainty measures of D-S theory are briefly described as follows:

Dubois and Prade’s weighted Hartley entropy is shown as follows [37]:

(10) $$H_{DP}\left(m\right)=-\sum_{A\subseteq 2^{\Theta }}m\left(A\right)\log\left(|A|\right).$$

where *A* is a focal element of Θ and |A| is the cardinality of *A*.

Höhle’s confusion measure is shown as follows [38]:

(11) $$H_{H}\left(A\right)=-\sum_{A\subseteq 2^{\Theta }}m\left(A\right)\log\left(Bel\left(A\right)\right).$$

The dissonance measure of Yager is defined as follows [39]:

(12) $$H_{Y}\left(A\right)=-\sum_{A\subseteq 2^{\Theta }}m\left(A\right)\log\left(Pl\left(A\right)\right).$$

The discord measure of Klir and Ramer is defined as follows [35]:

(13) $$H_{KR}\left(A\right)=-\sum_{A\subseteq 2^{\Theta }}m\left(A\right)\log\sum_{B\subseteq 2^{\Theta }}m\left(B\right)\frac{\left|A\cap B\right|}{\left|B\right|}.$$

where |B| is the cardinality of B, which is also a focal element of Θ.

Klir and Parviz gave their strife measure of entropy. It is defined as follows [40]:

(14) $$H_{KP}\left(A\right)=-\sum_{A\subseteq 2^{\Theta }}m\left(A\right)\log\sum_{B\subseteq 2^{\Theta }}m\left(B\right)\frac{\left|A\cap B\right|}{\left|A\right|}.$$

George and Pal proposed a method called conflict measure for entropy [51]:

(15) $$H_{GP}\left(A\right)=\sum_{A\subseteq 2^{\Theta }}m\left(A\right)\log\sum_{B\subseteq 2^{\Theta }}m\left(B\right)\left(1-\frac{\left|A\cap B\right|}{\left|A\cup B\right|}\right).$$

where |A∩B| and |A∪B| represent the cardinality of A∩B and A∪B, respectively.

### 2.3. Deng Entropy

For mass functions, a new uncertainty measure called Deng entropy under D-S framework was defined as follows [43]:

(16) $$E_{d}\left(m\right)=-\sum_{A\subseteq 2^{\Theta }}m\left(A\right)\log_{2}\frac{m(A)}{2^{\left|A\right|}-1}.$$

As shown in the above definition, Deng entropy is similar to the classical Shannon entropy in form. However, the mass function of each focal element is divided by a term ($2^{\left|A\right|}-1$), which means the scale of the focal element *A*. If each focal element is assigned only one element, Deng entropy will be degenerated to Shannon entropy as follows:

(17) $$E_{d}\left(m\right)=-\sum_{A\subseteq 2^{\Theta }}m\left(A\right)\log_{2}\frac{m(A)}{2^{\left|A\right|}-1}=-\sum_{A\subseteq 2^{\Theta }}m\left(A\right)\log_{2}m(A).$$

## 3. The Improved Belief Entropy

By reviewing the relative literatures, we conclude that the mass function, the belief function and the plausibility function are applicable to the mainstream measurement of uncertainty. Here, the belief interval [Bel(A),Pl(A)], which contains more information of D-S framework is often overlooked. There are only several articles about uncertainty measurement mentioning the belief interval [5,46,47,49,50,52]. In this article, an improved belief entropy involving the belief interval is proposed based on Deng entropy and the belief interval, defined as follows:

(18) $$\begin{array}{cc} H_{inter}\left(m\right)= & -\sum_{i=1}^{n}\frac{Bel\left(\theta_{i}\right)+Pl\left(\theta_{i}\right)}{2}\log_{2}\left(\frac{Bel\left(\theta_{i}\right)+Pl\left(\theta_{i}\right)}{2}e^{-(Pl(A)-Bel(A))}\right)\\ & -\sum_{A\neq \theta_{i},A\subseteq 2^{\Theta }}m\left(A\right)\log_{2}\left(\frac{m(A)}{2^{\left|A\right|}-1}e^{-(Pl(A)-Bel(A))}\right). \end{array}$$

The improved belief entropy proposed in this article has two improvements over Deng entropy. First, the mass function m(A) is used in Deng entropy while it is replaced by the central value of the belief interval [46]. Pl(A) and Bel(A) are the lower limit function and upper limit function of the probability to which proposition *A* is supported [53]. The belief interval has higher accuracy than the mass function in illustrating how strongly the evidence supports *A* under a strict D-S framework. For simplicity, the mean of Pl(A) and Bel(A) is employed to discretize the belief interval for computing. Additionally, a coefficient $e^{-(Pl(A)-Bel(A))}$ is added to the measurement of non-specificity of the belief structure. The non-specificity of the belief interval can be quantified by its imprecision degree, which is related to the span of the belief interval [49]. Additionally, the properties proposed by Klir and Wierman [54] for total uncertainty measurement of the improved belief entropy are explored as follows:

***Probabilistic consistency:*** If all the focal elements of a BPA are singletons, then m(x)=Bel(x)=Pl(x) for $\forall x\in 2^{\Theta }$. Obviously, the improved belief entropy will be degenerated to Shannon entropy. Hence, the probabilistic consistency property is verified.

***Set consistency:*** Set consistency requires that H(m)=log(|a|) whenever *m* is categorical with focal element *a*, i.e., m(a)=1. For the improved belief entropy, when m(a)=1:

(19) $$H_{inter}\left(m\right)=log_{2}\left(2^{\left|A\right|}-1\right)\geq log_{2}\left(|A|\right).$$

where |A| is the cardinality of *a*. Therefore, the belief entropy is not set consistent.

***Range:*** The range property requires that for any BPA $m_{X}$ in *X*, $0\leq H\left(m_{X}\right)\leq log_{2}\left(|X|\right)$. A simple counter example is employed:

Let $\Theta =\{\theta_{1},\theta_{2},\theta_{3},\theta_{4}\}$ be the FOD, for a mass function $m(\theta_{1},\theta_{2})=0.5$, $m(\theta_{3})=0.1$, $m(\theta_{2})=0.1$, and m(Θ)=0.3. Then,

$$\begin{array}{c} Bel\left(\theta_{1}\right)=0,Pl\left(\theta_{1}\right)=0.8,Bel\left(\theta_{1},\theta_{2}\right)=0.6,Pl\left(\theta_{1},\theta_{2}\right)=0.9,\\ Bel\left(\theta_{3}\right)=0.1,Pl\left(\theta_{3}\right)=0.6,Bel\left(\theta_{2}\right)=0.1,Pl\left(\theta_{2}\right)=0.9,\\ H_{inter}=-0.4log_{2}\left(0.4e^{-0.8}\right)-0.5log_{2}\left(0.5e^{-0.8}\right)-0.25log_{2}\left(0.25e^{-0.3}\right)\\ -0.5log_{2}\left(\frac{0.3}{7}e^{-0.3}\right)-0.3log_{2}\frac{0.3}{7}=5.5479,log_{2}3=1.5850<5.5479. \end{array}$$

Obviously, the improved belief entropy does not satisfy the property range.

***Subadditivity:*** To investigate whether the improved belief entropy verifies the subadditivity, we take the following example:

Let X×Y be the product space of the sets $X=\{x_{1},x_{2},x_{3}\}$ and $Y=\{y_{1},y_{2}\}$. The marginal BPAs on X×Y with masses is as follows:

$$m\left(\left\{z_{11},z_{12},z_{21}\right\}\right)=0.2,m\left(\left\{z_{31},z_{32}\right\}\right)=0.3,m\left(\left\{z_{21}\right\}\right)=0.1,m\left(X\times Y\right)=0.4.$$

where $z_{ij}=\left(x_{i},y_{i}\right)$, the marginal BPAs on *X* and *Y* are $m_{1}$ and $m_{2}$:

$$\begin{array}{c} m_{1}\left(x_{1},x_{2}\right)=0.2,m_{1}\left(x_{3}\right)=0.3,m_{1}\left(x_{2}\right)=0.1,m_{1}\left(X\right)=0.4,m_{2}\left(y_{1}\right)=0.1,m_{2}\left(Y\right)=0.9. \end{array}$$

Therefore,

$$\begin{array}{c} Bel\left(x_{1}\right)=0,Pl\left(x_{1}\right)=0.6,Bel\left(x_{1},x_{2}\right)=0.3,Pl\left(x_{1},x_{2}\right)=0.9,Bel\left(x_{3}\right)=0.3,Pl\left(x_{3}\right)=0.7,Bel\left(x_{2}\right)=0.1,\\ Pl\left(x_{2}\right)=0.7,Bel\left(y_{1}\right)=0.1,Pl\left(y_{1}\right)=1,Bel\left(\left\{z_{11},z_{12},z_{21}\right\}\right)=0.3,Pl\left(\left\{z_{11},z_{12},z_{21}\right\}\right)=0.7,\\ Bel\left(\left\{z_{11}\right\}\right)=Bel\left(\left\{z_{12}\right\}\right)=Bel\left(\left\{z_{22}\right\}\right)=Bel\left(\left\{z_{31}\right\}\right)=Bel\left(\left\{z_{32}\right\}\right)=0,Bel\left(\left\{z_{31},z_{32}\right\}\right)=0.3,\\ Pl\left(\left\{z_{11}\right\}\right)=Pl\left(\left\{z_{12}\right\}\right)=0.6,Pl\left(\left\{z_{31}\right\}\right)=Pl\left(\left\{z_{32}\right\}\right)=0.7,Pl\left(\left\{z_{31},z_{32}\right\}\right)=0.7,Bel\left(\left\{z_{21}\right\}\right)=0.1,\\ Pl\left(\left\{z_{21}\right\}\right)=0.7,Pl\left(\left\{z_{22}\right\}\right)=0.4,H_{inter}\left(m_{1}\right)+H_{inter}\left(m_{2}\right)=8.8473,H_{inter}\left(m\right)=8.8729. \end{array}$$

It is obvious that $H_{inter}\left(m_{1}\right)+H_{inter}\left(m_{2}\right)<H_{inter}\left(m\right)$. Hence, the property subadditivity is not satisfied.

***Additivity:*** To verify the property additivity, the notation of the last example is employed. Let X×Y be the product space of the sets $X=\{x_{1},x_{2},x_{3}\}$ and $Y=\{y_{1},y_{2}\}$. The marginal BPAs on X×Y with masses is as follows:

$$\begin{array}{c} m_{1}\left(x_{1},x_{2}\right)=0.2,m_{1}\left(x_{3}\right)=0.3,m_{1}\left(x_{2}\right)=0.1,m_{1}\left(X\right)=0.4,m_{2}\left(y_{1}\right)=0.1,m_{2}\left(Y\right)=0.9. \end{array}$$

The following BPA $m^{\prime }=m^{1}\times m^{2}$ on X×Y is built. The marginal BPAs of $m^{\prime }$ are $m^{1}$ and $m^{2}$, which are noninteractive. The masses of $m^{\prime }$ are as follows:

$$m^{\prime }\left(\left\{z_{11},z_{12},z_{21}\right\}\right)=0.2,m^{\prime }\left(\left\{z_{31},z_{32}\right\}\right)=0.3,m^{\prime }\left(\left\{z_{21}\right\}\right)=0.1,m^{\prime }\left(X\times Y\right)=0.4.$$

where $z_{ij}=\left(x_{i},y_{i}\right)$. It can be calculated:

$$H_{inter}\left(m_{1}\right)+H_{inter}\left(m_{2}\right)=8.8473,H_{inter}\left(m^{\prime }\right)=8.8729.$$

The result that $H_{inter}\left(m_{1}\right)+H_{inter}\left(m_{2}\right)<H_{inter}\left(m^{\prime }\right)$ shows that the property additivity is not satisfied. In summary, the improved entropy satisfies probabilistic consistency and set consistency but does not satisfy property range, additivity and subadditivity. It is true that these five requirements are helpful to identify whether a definition of the belief entropy makes sense. However, they are not the only criteria for judging rationality and effectiveness of a measurement of belief entropy. On the one hand, these requirements are motivated by the properties of Shannon entropy, which is not entirely applicable for D-S theory framework and there is no uniform definition of uncertainty measurement in D-S theory currently. It should be tolerable that there are very few measurements of uncertainty under D-S framework satisfying all the requirements. They deserve the opportunity to be tested in practice and it is true of the improved belief entropy in this paper. On the other hand, the properties of the belief entropy should keep pace with the times. It was twenty years ago that Klir and Wierman proposed these five requirements. Since then, there are significant increments in research on measurements of uncertainty in D-S theory. Some shortcomings in the properties of Klir and Wierman were stated by Radim and Prakash [55] and they proposed a list of six desired properties of entropy for D-S theory, which are different from those of Klir and Wierman. None of the existing definitions satisfy these six properties except itself. Hence, the properties of measurements of uncertainty in D-S theory need further research.

## 4. Numerical Examples

In this section, several examples are employed to show the efficiency of the improved belief entropy.

### 4.1. Example 1

Let $\Theta =\{\theta_{1},\theta_{2},\ldots ,\theta_{n}\}$ be the FOD. There is a vacuous BPA m(Θ)=1 under the FOD. It is obvious that:

Bel(Θ)=Pl(Θ)=1.

The associated Shannon entropy and the improved belief entropy of the FOD are as follows:

$$H_{s}\left(m\right)=-1\times log_{2}1=0,H_{inter}=-1\times log_{2}{\left(2^{n}-1\right)}^{-1}=log_{2}\left(2^{n}-1\right).$$

Again, the above results verify that the improved belief entropy will deteriorate to Shannon entropy when there is only a single element in the vacuous BPA. $H_{inter}>H_{s}$ if n>1 and $H_{inter}\to n$ when n→+∞. The result which is the same as Deng entropy is logical because uncertainty will increase as the number of elements in the vacuous BPA increases. It can also be seen that there are limitations of the application of Shannon entropy in D-S theory.

### 4.2. Example 2

Given a FOD $\Theta =\{\theta_{1},\theta_{2},\theta_{3},\theta_{4},\theta_{5}\}$, $m(\theta_{i})=0.2$ for i=1,2,3,4,5. Then, $Bel\left(\theta_{i}\right)=Bl\left(\theta_{i}\right)=0.2$ for i=1,2,3,4,5. The associated Shannon entropy and the improved belief entropy of the FOD are as follows:

$$\begin{array}{c} H_{s}\left(m\right)=-0.2\times log_{2}0.2\times 5=2.3219,\\ H_{inter}\left(m\right)=-\frac{0.2+0.2}{2}log_{2}\left(\frac{0.2+0.2}{2(2^{1}-1)}e^{-{\left(0.2-0.2\right)}^{2}}\right)\times 5=2.3219. \end{array}$$

It is obvious that Shannon entropy is equal to the improved belief entropy in this example, which verifies that the improved belief entropy is probability consistent if BPA is Bayesian.

### 4.3. Example 3

Let *m* be a belief structure in Θ={a,b,c,d}; there are two cases of BPAs in this FOD: $m_{1}\left(\left\{a,b\right\}\right)=0.4$, $m_{1}\left(\left\{c,d\right\}\right)=0.6$ and $m_{2}\left(\left\{a,c\right\}\right)=0.4$,$m_{2}\left(\left\{b,c\right\}\right)=0.6$. The result with Deng Entropy is as follows [4]:

$$\begin{array}{cc} \; & E_{d}\left(m_{1}\right)=-0.4log_{2}\frac{0.4}{2^{2}-1}-0.6log_{2}\frac{0.6}{2^{2}-1}=2.5559,\\ \; & E_{d}\left(m_{2}\right)=-0.4log_{2}\frac{0.4}{2^{2}-1}-0.6log_{2}\frac{0.6}{2^{2}-1}=2.5559. \end{array}$$

The limitations of Deng entropy are transparent because the result calculated by Deng entropy is counterintuitive. The mass values of the two BPAs are the same, while the FOD of the first BPA $m_{1}$ consists of four events a,b,c,d and $m_{2}$ consists of three events a,b,c. Intuitively, the uncertainty of $m_{2}$ should be less than $m_{1}$. However, the result that Deng entropy of both BPAs is equivalent illustrates that Deng entropy does not recognize the difference between $m_{1}$ and $m_{2}$.

With the improved belief entropy, the result is calculated as follows:

$$\begin{array}{c} H_{inter}\left(m_{1}\right)=-0.4log_{2}\left(0.2e^{-0.4}\right)-0.6log_{2}\left(0.3e^{-0.6}\right)-0.4log_{2}\frac{0.4}{3}-0,6log_{2}0.2=5.9696,\\ H_{inter}\left(m_{2}\right)=-0.2log_{2}\left(0.2e^{-0.4}\right)-0.3log_{2}\left(0.3e^{-0.6}\right)-0.5log_{2}\left(0.5e^{-1}\right)\\ -0.4log_{2}\left(0.4e^{-0.6}\right)-0.6log_{2}\left(0.6e^{-0.4}\right)=5.8303. \end{array}$$

The experimental results show that the improved belief entropy of $m_{2}$ is less than $m_{1}$. It can be concluded that the improved belief entropy can effectively measure the difference between these two BPAs by taking more reliable information implied in different BPAs into consideration.

### 4.4. Example 4

Let FOD Θ be $\left\{\theta_{1},\theta_{2}\right\}$ and assume that there are two BPAs $m_{1}$ and $m_{2}$ over Θ:

$$\begin{array}{c} m_{1}\left(\left\{\theta_{2}\right\}\right)=0.8,m_{1}\left(\left\{\theta_{1},\theta_{2}\right\}\right)=0.2,m_{2}\left(\left\{\theta_{1}\right\}\right)=0.2,m_{2}\left(\left\{\theta_{2}\right\}\right)=0.8. \end{array}$$

A comparative experiment is conducted. Another uncertainty measure SU also based on the belief interval is introduced [49]. The definition of SU is shown as follows:

Let *m* be a BPA defined on the FOD $\Theta =\{\theta_{1},\theta_{2},\ldots ,\theta_{i},\ldots ,\theta_{N}\}$. The total uncertainty degree of *m* can be expressed by

$$SU\left(m\right)=\sum_{i=1}^{n}\left[-\frac{Bel\left(\left\{\theta_{i}\right\}\right)+Pl\left(\left\{\theta_{i}\right\}\right)}{2}log_{2}\frac{Bel\left(\left\{\theta_{i}\right\}\right)+Pl\left(\left\{\theta_{i}\right\}\right)}{2}+\frac{Bel\left(\left\{\theta_{i}\right\}\right)-Pl\left(\left\{\theta_{i}\right\}\right)}{2}\right].$$

For $m_{1}$:

$$\begin{array}{c} \left[Bel_{m_{1}}\left(\left\{\theta_{1}\right\}\right),Pl_{m_{1}}\left(\left\{\theta_{1}\right\}\right)\right]=\left[0,0.2\right],\\ \left[Bel_{m_{1}}\left(\left\{\theta_{2}\right\}\right),Pl_{m_{1}}\left(\left\{\theta_{2}\right\}\right)\right]=\left[0.8,1\right],\\ \left[Bel_{m_{1}}\left(\left\{\theta_{1},\theta_{2}\right\}\right),Pl_{m_{1}}\left(\left\{\theta_{1},\theta_{2}\right\}\right)\right]=\left[1,1\right]. \end{array}$$

For $m_{2}$:

$$\begin{array}{c} \left[Bel_{m_{2}}\left(\left\{\theta_{1}\right\}\right),Pl_{m_{2}}\left(\left\{\theta_{1}\right\}\right)\right]=\left[0.2,0.2\right],\\ \left[Bel_{m_{2}}\left(\left\{\theta_{2}\right\}\right),Pl_{m_{2}}\left(\left\{\theta_{2}\right\}\right)\right]=\left[0.8,0.8\right],\\ \left[Bel_{m_{2}}\left(\left\{\theta_{1},\theta_{2}\right\}\right),Pl_{m_{2}}\left(\left\{\theta_{1},\theta_{2}\right\}\right)\right]=\left[1,1\right]. \end{array}$$

Thus,

$$\forall A\subseteq \Theta :\left[Bel_{m_{1}}\left(A\right),Pl_{m_{1}}\left(A\right)\right]⊇\left[Bel_{m_{2}}\left(A\right),Pl_{m_{2}}\left(A\right)\right]$$

The results are as follows:

$$\begin{array}{c} SU\left(m_{1}\right)=-\frac{0+0.2}{2}log_{2}\frac{0+0.2}{2}+\frac{0.2-0}{2}+\left(-\frac{0.8+1}{2}log_{2}\frac{0.8+1}{2}\right)=0.6690,\\ SU\left(m_{2}\right)=-0.2log_{2}0.2-0.8log_{2}0.8=0.7219. \end{array}$$

It is obvious that $SU\left(m_{1}\right)<SU\left(m_{2}\right)$. This result shows that the monotonicity defined by Abellan [56] between $m_{1}$ and $m_{2}$ is violated by the uncertainty measure SU. The property of monotonicity is defined as follows:

There is an uncertainty measure UM and two arbitrary BPAs $m_{1}$ and $m_{2}$ over the FOD Θ. UM satisfies the property monotonicity if

(20) $$\forall A\subseteq \Theta :\left[Bel_{m_{1}}\left(A\right),Pl_{m_{1}}\left(A\right)\right]\subseteq \left[Bel_{m_{2}}\left(A\right),Pl_{m_{2}}\left(A\right)\right].$$

$UM\left(m_{1}\right)\leq UM\left(m_{2}\right)$ exists.

With the improved belief entropy, the result of the example above is calculated as follows:

$$\begin{array}{c} H_{inter}\left(m_{1}\right)=-0.1log_{2}\left(0.4e^{-0.2}\right)-0.9log_{2}\left(0.9e^{-0.2}\right)-0.2log_{2}\frac{0.2}{3}=1.5389,\\ H_{inter}\left(m_{2}\right)=-0.2log_{2}0.2-0.8log_{2}0.8=0.7219. \end{array}$$

The result that $H_{inter}\left(m_{1}\right)>H_{inter}\left(m_{2}\right)$ shows the improved belief entropy performs better than SU over this example in terms of the property monotonicity. The rigorous proof of the property monotonicity need further research.

### 4.5. Example 5

There is a FOD Θ={1,2,…,14,15} with 15 elements. The BPA of Θ is as follows:

m({7})=0.05,m({3,4,5})=0.05,m({A})=0.8,m({Θ})=0.1.

The number of elements in the proposition *A* changes from 1 to 14. To verify the advantages of the improved belief entropy, another eight uncertainty measures are introduced for comparison: Deng entropy [43], Höhle’s confusion measure [38], Yager’s dissonance measure [39], Dubois and Prade’s weighted Hartley entropy [37], Klir and Ramer’s discord measure [35], Klir and Parviz’s strife measure [40], George and Pal’s conflict measure [51], Pan and Zhou’s measure [50] and Zhou et al.’s measure [4].

**Figure 1:**
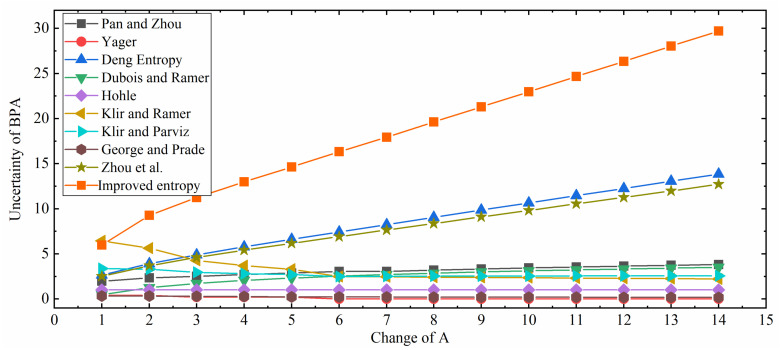
Comparison among different uncertainty measures (Pan and Zhou [50], Yager [39], Deng Entropy [43], Dubois and Prade [37], Höhle [38], Klir and Ramer [35], Klir and Parviz [40], George and Pal [51], Zhou et al. [4], and Improved entropy).

**Figure 2:**
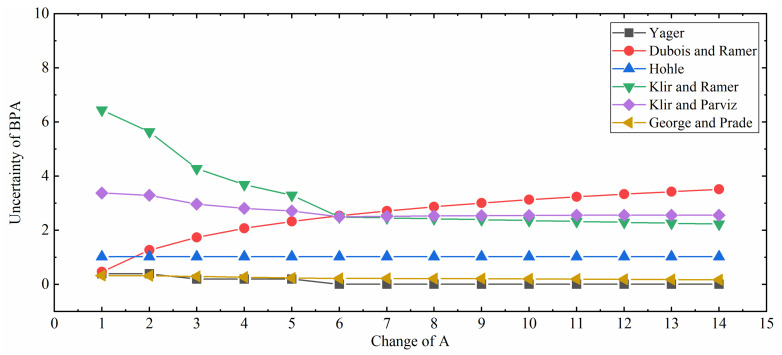
Comparison among different uncertainty measures (Yager [39], Dubois and Prade [37], Höhle [38], Klir and Ramer [35], Klir and Parviz [40], and George and Pal [51]).

The experimental results are shown in Table 1 and the results of these uncertainty measures are plotted in Figure 1. To make it more visible and understandable, the results of the methods in [35,37,38,39,40,51] are extracted and plotted in Figure 2. These curves can be divided into two categories. The first category includes Höhle’s confusion measure, Yager’s dissonance measure, Klir and Ramer’s discord measure, Klir and Parviz’s strife measure, and George and Pal’s conflict measure. They are on a downward trend with the rising of the element number of *A* or flat, which is counterintuitive because these measurements only measure the discord uncertainty while ignore the non-specificity uncertainty. The other category consists of Deng entropy, Pan and Zhou’s measure, Dubois and Prade’s weighted Hartley entropy and the improved belief entropy proposed in this paper. What they have in common is that they are on a rising trend with the increment of the element number in *A*. These methods also take non-specificity into consideration so their results are rational. However, Dubois and Prade’s weighted Hartley entropy only considers non-specificity while ignoring discord uncertainty. Deng entropy fails to detect the BPAs of which an element belongs to different focal elements. Pan and Zhou’s method applies the pignistic transformation and the plausibility transformation while they have deficiencies and limitations in D-S theory. Compared with Zhou et al.’s measure, which is a fairly comprehensive uncertainty measure of BPA, the improved belief entropy follows the same trend. Therefore, the improved belief entropy is relatively more effective and reasonable compared with other uncertainty measures under D-S theory framework, both considering the central value and the span of the belief interval.

## 5. Application

To verify the effectiveness and the applicability of the improved belief entropy in practice, the case study in [57] and the fault diagnosis method in [44] are employed in this part. The difference is that this paper represents Deng entropy applied in [44] with the improved belief entropy for comparison.

The problem is described as follows. There are three fault types denoted as $F_{1}$, $F_{2}$, and $F_{3}$. The FOD is $\Theta =\{F_{1},F_{2},F_{3}\}$. Three sensor reports of the diagnostic result are listed in Table 2. With Dempster’s rule of combination in Equation (6), the combination result is shown in Table 3. It can be seen that it is difficult to judge which fault type has occurred because the BPA of $F_{1}$ and $F_{2}$ after combination is very close. Dempster’s rule of combination does not play a part in this case.

To solve this problem, a fault diagnosed method based on Deng entropy [44] is put forward. The uncertainty or reliability of sensor data will be modeled as a weight of each BPA, which is defined as follows:

(21) $$w\left(i\right)=w_{s}\left(i\right)\times w_{d}\left(i\right).$$

where $w_{s}\left(i\right)$ means the static reliability and $w_{d}\left(i\right)$ represents the dynamic reliability. $w_{s}\left(i\right)$ of each BOE is listed in Table 4. $w_{d}\left(i\right)$ is defined as follows:

(22) $$w_{d}\left(i\right)=Crd\left(i\right)\times \frac{E_{d}\left(m_{i}\right)}{max\{E_{d}\left(m_{i}\right)\}}.$$

where Crd(i) is the credibility degree of $E_{i}$. $E_{d}\left(m_{i}\right)$ is the Deng entropy of $E_{i}$. $max\{E_{d}\left(m_{i}\right)\}$ represents the maximum of all the $E_{d}\left(m_{i}\right)$. Crd(i) and $E_{d}\left(m_{i}\right)$ of three BOEs are shown in Table 4. Details can be found in the work of Yuan et al. [44]. The ultimate weight of each BOE based on the improved belief entropy $H_{inter}\left(m_{i}\right)$ proposed in this paper is defined as follows:

(23) $$w\left(i\right)=w_{s}\left(i\right)\times Crd\left(i\right)\times \frac{max\{H_{inter}\left(m_{i}\right)\}}{H_{inter}\left(m_{i}\right)}.$$

It can be seen that Equation (23) is a little different from that in [44]. Intuitively, a piece of evidence with less uncertainty should be endowed with higher weight, which is consistent with the principle of the entropy weight method. Therefore, the method in [44] is modified and replaced with Equation (23). The improved belief entropy of each BOE is shown as follows:

$$\begin{array}{cc} H_{inter}\left(m_{1}\right)= & -\sum_{i=1}^{n}\frac{Bel_{1}\left(\theta_{i}\right)+Pl_{1}\left(\theta_{i}\right)}{2}\log_{2}\left(\frac{Bel_{1}\left(\theta_{i}\right)+Pl_{1}\left(\theta_{i}\right)}{2}e^{-(Pl_{1}\left(A\right)-Bel_{1}\left(A\right))}\right)\\ & -\sum_{A\neq \theta_{i},A\subseteq 2^{\Theta }}m\left(A\right)\log_{2}\left(\frac{m(A)}{2^{\left|A\right|}-1}e^{-(Pl_{1}\left(A\right)-Bel_{1}\left(A\right))}\right)\\ & =-0.7\log_{2}\left(0.7e^{-0.2}\right)-0.25\log_{2}\left(0.25e^{-0.3}\right)-0.15\log_{2}\left(0.15e^{-0.3}\right)\\ & -0.1\log_{2}\left(\frac{0.1}{3}e^{-0.2}\right)-0.2\log_{2}\frac{0.2}{7}\\ & =3.1912, \end{array}$$

$$\begin{array}{cc} H_{inter}\left(m_{2}\right)= & -\sum_{i=1}^{n}\frac{Bel_{2}\left(\theta_{i}\right)+Pl_{2}\left(\theta_{i}\right)}{2}\log_{2}\left(\frac{Bel_{2}\left(\theta_{i}\right)+Pl_{2}\left(\theta_{i}\right)}{2}e^{-(Pl_{2}\left(A\right)-Bel_{2}\left(A\right))}\right)\\ & -\sum_{A\neq \theta_{i},A\subseteq 2^{\Theta }}m\left(A\right)\log_{2}\left(\frac{m(A)}{2^{\left|A\right|}-1}e^{-(Pl_{2}\left(A\right)-Bel_{2}\left(A\right))}\right)\\ & =-0.1\log_{2}\left(0.1e^{-0.1}\right)-0.875\log_{2}\left(0.875e^{-0.15}\right)-0.075\log_{2}\left(0.075e^{-0.15}\right)\\ & -0.05\log_{2}\left(\frac{0.05}{3}e^{-0.1}\right)-0.1\log_{2}\frac{0.1}{7}\\ & =1.9165,\\ H_{inter}\left(m_{3}\right)= & -\sum_{i=1}^{n}\frac{Bel_{3}\left(\theta_{i}\right)+Pl_{3}\left(\theta_{i}\right)}{2}\log_{2}\left(\frac{Bel_{3}\left(\theta_{i}\right)+Pl_{3}\left(\theta_{i}\right)}{2}e^{-(Pl_{3}\left(A\right)-Bel_{3}\left(A\right))}\right)\\ & -\sum_{A\neq \theta_{i},A\subseteq 2^{\Theta }}m\left(A\right)\log_{2}\left(\frac{m(A)}{2^{\left|A\right|}-1}e^{-(Pl_{3}\left(A\right)-Bel_{3}\left(A\right))}\right)\\ & =-0.75\log_{2}\left(0.75e^{-0.1}\right)-0.2\log_{2}\left(0.2e^{-0.2}\right)-0.1\log_{2}0.1e^{-0.2}-0.1\log_{2}\left(\frac{0.1}{3}e^{-0.1}\right)\\ & -0.1\log_{2}\frac{0.1}{7}\\ & =2.4207. \end{array}$$

The weight of each BOE based on the improved belief entropy is calculated as follows:

$$\begin{array}{c} w\left(1\right)=w_{s}\left(1\right)\times Crd\left(1\right)\times \frac{H_{inter}\left(m_{1}\right)}{H_{inter}\left(m_{1}\right)}=1\times 1\times \frac{3.1912}{3.1912}=1,\\ w\left(2\right)=w_{s}\left(2\right)\times Crd\left(2\right)\times \frac{H_{inter}\left(m_{1}\right)}{H_{inter}\left(m_{2}\right)}=0.2040\times \;0.5523\times \frac{3.1912}{1.9165}=0.1876,\\ w\left(3\right)=w_{s}\left(3\right)\times Crd\left(3\right)\times \frac{H_{inter}\left(m_{1}\right)}{H_{inter}\left(m_{3}\right)}=1\times \;0.9660\times \frac{3.1912}{2.4207}=1.2735. \end{array}$$

The final weight of each BOE after normalization is shown as follows:

$$\begin{array}{c} w^{\prime }\left(1\right)=\frac{w(1)}{w(1)+w(2)+w(3)}=0.4063,\\ w^{\prime }\left(2\right)=\frac{w(2)}{w(1)+w(2)+w(3)}=0.0762,\\ w^{\prime }\left(3\right)=\frac{w(3)}{w(1)+w(2)+w(3)}=0.5175. \end{array}$$

The BPA of each proposition is modified by the final weight as follows:

$$\begin{array}{c} m(\left\{F_{1}\right\})=0.4063\times 0.6+0.0762\times 0.05+0.5175\times 0.7=0.6099,\\ m(\left\{F_{2}\right\})=0.4063\times 0.1+0.0762\times 0.8+0.5175\times 0.1=0.1533,\\ m(\left\{F_{2},F_{3}\right\})=0.4063\times 0.1+0.0762\times 0.05+0.5175\times 0.10=0.0962,\\ m(\Theta )=0.4063\times 0.2+0.0762\times 0.10+0.5175\times 0.10=0.1406. \end{array}$$

The fused result of the weighted BPA with the Dempster’s rule of combination is calculated as follows:

$$\begin{array}{c} m^{\prime \prime }\left(\left\{F_{1}\right\}\right)=\left(m⊕m\right)⊕m(\left(\left\{F_{1}\right\}\right)=0.8763,\\ m^{\prime \prime }\left(\left\{F_{2}\right\}\right)=\left(m⊕m\right)⊕m(\left(\left\{F_{2}\right\}\right)=0.0961,\\ m^{\prime \prime }\left(\left\{F_{2},F_{3}\right\}\right)=\left(m⊕m\right)⊕m(\left(\left\{F_{2},F_{3}\right\}\right)=0.0219,\\ m^{\prime \prime }\left(\Theta \right)=\left(m⊕m\right)⊕m\left(\Theta \right)=0.0057. \end{array}$$

The fused results with the improved belief entropy based on Dempster’s rule of combination are compared with several other methods, as shown in Table 5. Intuitively, $F_{1}$ should be the fault type that occurred because both $E_{1}$ and $E_{3}$ have relatively strong support to $F_{1}$ (0.60 and 0.70) while $E_{2}$ may come from an abnormal sensor compared with other two BOEs. The support of $F_{1}$ with Yuan et al.’s method based on the improved belief entropy is as high as Fan et al.’s method, Yuan et al.’s method and Zhou et al.’s method and the result is that $F_{1}$ is the fault type occurred. This case study verifies the applicability of the improved belief entropy.

## 6. Conclusions

The measurement of uncertainty under D-S theory framework is still an open issue. An improved belief entropy, which takes the central value and the span of the belief interval into consideration together when defining the uncertainty measure of BPA, is proposed based on Deng entropy and the belief interval in this paper. Importantly, as an uncertainty measure of BPA, it will degenerate to Shannon entropy when BPA is Bayesian, which is consistent with previous methods. Several numerical examples are conducted to verify the efficiency and flexibility of the improved belief entropy. The results of these examples show that the improved belief entropy performs better compared with other methods. To verify the applicability, a case study about fault diagnose is employed. The improved belief entropy will provide an insight to measure uncertainty in various fields (e.g., decision making, risk analysis, and pattern recognition) and further information processing. Although this study shows promising results, some limitations are worth consideration. First, the formula of the improved belief entropy is relatively complex, which leads to a high computational burden when faced with a large amount of evidences. Second, some critical properties (e.g., monotonicity) of the improved belief entropy are not proved by the study. Given the limitations of this study, future research is necessary to simplify the formula and investigate the critical properties of the improved belief entropy.

## Figures and Tables

**Table 1:** The results of different uncertainty measures.

Cases	Pan and Zhou	Yager	Deng Entropy	Dubois and Prade	Höhle	Klir and Ramer	Klir and Parviz	George and Pal	Zhou et al.	The Improved Belief Entropy
A = {1}	1.9757	0.3952	2.6623	0.4699	1.0219	6.4419	3.3804	0.3317	2.5180	5.9870
A = {1, 2}	2.3362	0.3952	3.9303	1.2699	1.0219	5.6419	3.2956	0.3210	3.7090	9.2881
A = {1, 2, 3}	2.5232	0.1997	4.9082	1.7379	1.0219	4.2823	2.9709	0.2943	4.6100	11.2461
A = {1, 2, 3, 4}	2.7085	0.1997	5.7878	2.0699	1.0219	3.6863	2.8132	0.2677	5.4127	12.9904
A = {1, 2, 3, 4, 5}	2.8749	0.1997	6.6256	2.3274	1.0219	3.2946	2.7121	0.2410	6.1736	14.6352
A = {1, 2,..., 6}	3.0516	0.0074	7.4441	2.5379	1.0219	2.4888	2.4992	0.2250	6.9151	16.3330
A = {1, 2,..., 7}	3.0647	0.0074	8.2532	2.7158	1.0219	2.4562	2.5198	0.2219	7.6473	17.9447
A = {1, 2,..., 8}	3.2042	0.0074	9.0578	2.8699	1.0219	2.4230	2.5336	0.2170	8.3749	19.6287
A = {1, 2,..., 9}	3.3300	0.0074	9.8600	3.0059	1.0219	2.3898	2.5431	0.2108	9.1002	21.3103
A = {1, 2,..., 10}	3.4445	0.0074	10.6612	3.1275	1.0219	2.3568	2.5494	0.2037	9.8244	22.9908
A = {1, 2,..., 11}	3.5497	0.0074	11.4617	3.2375	1.0219	2.3241	2.5536	0.1959	10.5480	24.6708
A = {1, 2,..., 12}	3.6469	0.0074	12.2620	3.3379	1.0219	2.2920	2.5562	0.1877	11.2714	26.3504
A = {1, 2,..., 13}	3.7374	0.0074	13.0622	3.4303	1.0219	2.2605	2.5577	0.1791	11.9946	28.0300
A = {1, 2,..., 14}	3.8219	0.0074	13.8622	3.5158	1.0219	2.2296	2.5582	0.1701	12.7177	29.7094

**Table 2:** BPAs of the sensors report.

Sensors Report	{*F*_1_}	{*F*_2_}	{*F*_2_,*F*_3_}	Θ
$E_{1}:m_{1}\left(\cdot \right)$	0.60	0.10	0.10	0.20
$E_{2}:m_{2}\left(\cdot \right)$	0.05	0.80	0.05	0.10
$E_{3}:m_{3}\left(\cdot \right)$	0.70	0.10	0.10	0.10

**Table 3:** Fused results of the sensors report (Dempster’s rule of combination).

	*F*_1_	*F*_2_	*F*_2_,*F*_3_	Θ
Fused Results	0.4519	0.5048	0.0336	0.0096

**Table 4:** $w_{s}\left(i\right),Crd\left(i\right),E_{d}\left(i\right)$ of three BOEs.

	*E*_1_	*E*_2_	*E*_3_
$w_{s}\left(i\right)$	1.0000	0.2040	1.0000
Crd(i)	1.0000	0.5523	0.9660
$E_{d}\left(i\right)$	2.2909	1.3819	1.7960

**Table 5:** The comparison of the fused results among different methods.

Methods	{*F*_1_}	{*F*_2_}	{*F*_2_,*F*_3_}	{Θ}
Dempster’s rule of combination [11,12]	0.4519	0.5048	0.0336	0.0096
Fan et al.’s method [57]	0.8119	0.1096	0.0526	0.0259
Yuan et al.’s method [44]	0.8948	0.0739	0.0241	0.0072
Zhou et al.’s method [58]	0.8951	0.0738	0.0240	0.0071
The improved belief entropy	0.8763	0.0961	0.0219	0.0057
